# Associations between self-reported pest treatments and pesticide concentrations in carpet dust

**DOI:** 10.1186/s12940-015-0015-x

**Published:** 2015-03-25

**Authors:** Nicole C Deziel, Joanne S Colt, Erin E Kent, Robert B Gunier, Peggy Reynolds, Benjamin Booth, Catherine Metayer, Mary H Ward

**Affiliations:** Yale School of Public Health, Department of Environmental Health Sciences, 60 College St., New Haven, CT 06510 USA; Occupational and Environmental Epidemiology Branch, Division of Cancer Epidemiology and Genetics, National Cancer Institute, National Institutes of Health, 9609 Medical Center Dr., Rockville, MD 20850 USA; Outcomes Research Branch, Applied Research Program, Division of Cancer Control and Population Sciences, National Cancer Institute, National Institutes of Health, 9609 Medical Center Dr., Rockville, MD 20850 USA; School of Public Health, University of California, 50 University Hall, Berkeley, CA 94720 USA; Cancer Prevention Institute of California, 2201 Walnut Ave., Fremont, CA 94538 USA; Department of Health Research and Policy, Stanford University School of Medicine, 291 Campus Dr., Stanford, CA 94305 USA

**Keywords:** Childhood leukemia, Dust, Exposure assessment, Environmental epidemiology, Pesticides, Questionnaire validation

## Abstract

**Background:**

Recent meta-analyses demonstrate an association between self-reported residential pesticide use and childhood leukemia risk. Self-reports may suffer from recall bias and provide information only on broad pesticide categories. We compared parental self-reported home and garden pest treatments to pesticides measured in carpet dust.

**Methods:**

Parents of 277 children with leukemia and 306 controls in Northern and Central California (2001–2007) were asked about insect and weed treatments during the previous year. Carpet dust samples were analyzed for 47 pesticides. We present results for the 7 insecticides (carbaryl, propoxur, chlorpyrifos, diazinon, cyfluthrin, cypermethrin, permethrin), 5 herbicides (2,4-dichlorophenoxyacetic acid [2,4-D], chlorthal, dicamba, mecoprop, simazine), and 1 synergist (piperonyl butoxide) that were present in home and garden products during the study period and were detected in ≥25% of carpet dust samples. We constructed linear regression models for the relative change in pesticide concentrations associated with self-reported treatment of pest types in cases and controls separately and combined, adjusting for demographics, housing characteristics, and nearby agricultural pesticide applications.

**Results:**

Several self-reported treatments were associated with pesticide concentrations in dust. For example, households with flea/tick treatments had 2.3 (95% Confidence Interval [CI]: 1.4, 3.7) times higher permethrin concentrations than households not reporting this treatment. Households reporting treatment for ants/cockroaches had 2.5 (95% CI: 1.5, 4.2) times higher cypermethrin levels than households not reporting this treatment. Weed treatment by a household member was associated with 1.9 (1.4, 2.6), 2.2 (1.6, 3.1), and 2.8 (2.1, 3.7) times higher dust concentrations of dicamba, mecoprop, and 2,4-D, respectively. Weed treatments by professional applicators were null/inversely associated with herbicide concentrations in dust. Associations were generally similar between cases and controls and were consistent with pesticide active ingredients in these products during the study time period.

**Conclusions:**

Consistency between self-reported pest treatments, concentrations in dust, and pesticides in products lends credibility to the exposure assessment methods and suggests that differential recall by case–control status is minimal.

**Electronic supplementary material:**

The online version of this article (doi:10.1186/s12940-015-0015-x) contains supplementary material, which is available to authorized users.

## Introduction

Recent meta-analyses support a link between self-reported residential pesticide exposure and increased risk of childhood leukemia [1-3]. Epidemiologic studies have mostly relied on parental self-reports to broadly characterize types of pesticides used (e.g., insecticides, herbicides) and the timing of exposure (e.g., pre-conception, prenatal, early childhood). Self-reports may be subject to inaccurate recall or recall bias and generally do not provide information on specific active ingredients [4-6]. Improved exposure assessment methods are needed to confirm associations observed with self-reported pesticide use and to identify specific etiologic agents.

Pesticide measurements in carpet dust may serve as a useful, objective indicator of past exposures. The measurements are independent of recall, and the dust can be analyzed for numerous pesticide active ingredients (“pesticides”). Dust is a reservoir for chemicals in the home and is an important source of pesticide exposure for children. Non-dietary ingestion of dust has been estimated to contribute up to 40% of total exposure in children, depending on the pesticide [7-9], due to the high percentage of time children spend indoors and on the floor as well as their propensity to engage in hand-to-mouth activity [10,11]. In addition, pesticide concentrations in a single carpet dust sample may represent exposure over months or years, because pesticides resist degradation due to limited exposure to sunlight, microbial activity, moisture, and other factors [10,11]. Measurements of several home and garden pesticides in dust samples collected within the same California homes over a period of approximately 2 years exhibited moderate to strong correlation [12].

In the initial phase (1999–2002) of the Northern California Childhood Leukemia Study (NCCLS), a population-based case–control study, parental self-reported insect treatments and treatment of lawns, weeds, and other outdoor plants were associated with an increased risk of childhood leukemia [13]. In the present analysis, we compared self-reported pest treatments and concentrations of pesticides in residential dust samples from a subset of homes of cases and controls interviewed in 2001 to 2007. The objectives of this analysis were to (1) identify associations between self-reported pest treatments and pesticides in residential dust, (2) compare findings for cases and controls to assess differences in these associations, and (3) determine whether these associations were consistent with known uses of these pesticides.

## Methods

### Study population and design

The design of the NCCLS has been described previously [14,15]. Briefly, children under the age of 15 years diagnosed with leukemia from 1995–2008 were enrolled from nine major pediatric clinical centers in 35 counties in the San Francisco Bay Area and the Central Valley. Controls were selected randomly from the California birth registry (Sacramento, CA) and individually matched on the child’s date of birth, sex, and Hispanic ethnicity, and mother’s race. Interviews of the child’s primary caregiver (98% the mother) were conducted in the home; information initially collected included (but was not limited to) demographics, home and garden pest treatments, and occupational histories for both caregivers/parents. From December 1999 to June 2006, cases and controls who were <8 years old at diagnosis (or a corresponding reference date for the matched controls) and still living at the diagnosis/reference home were eligible for a second home visit during which the study team collected residential dust samples, detailed information on home and garden pesticide use [16], and an inventory of pesticide products stored in the home [17]. Eligibility was limited to younger cases and controls so that the dust sample would reflect exposures over a substantial portion of the child’s early life. Of the 731 eligible households, 296 cases (91%) and 333 (82%) controls participated in the second interview (2001–2007). Among those, 277 cases (94%) and 306 controls (92%) had adequate dust for at least one chemical analysis method. All research was conducted in accordance with requirements of the institutional review boards at the University of California, Berkeley, the California State Committee for the Protection of Human Subjects, the National Cancer Institute (NCI), and participating hospitals and institutions. All participants provided informed consent.

### Self-reported pest treatments

At the time of dust collection at the second home visit, interviewers asked the 583 biological parents/caregivers if pesticides were used in and around the home and garden in the previous 12 months. Because of the longer time needed to enroll controls in the main study, the duration between diagnosis/reference and dust sampling was shorter for cases (median years [inter-quartile range]: 0.9 [0.7–1.3]) than for controls (1.7 [1.3–2.2]). We queried about treatment of ants/cockroaches, carpenter ants/termites, fleas/ticks in home, fleas/ticks on pets, flea/tick shampoo, flea/tick collar, bees/wasps/hornets, flies/mosquitos, indoor plants, lawn/garden insects, lawn/garden weeds, use of a fogger/bomb product, professional inside treatments, professional outdoor insect treatments (including to the foundation, exterior, or lawn), and professional treatment of lawn/garden weeds.

A subset of parents/caregivers (241 cases and 255 controls) were also asked about pest treatments during the 3 months pre-conception through birth (i.e., 12 months before birth) and the first three years after the child’s birth.^13^ These questions replicated those asked in the first interview and there was moderate to high reliability between responses to these questions and the earlier reports with Kappa statistics ranging from 0.31 to 0.61 [16].

### Residential dust collection

As described previously [14,18], from October 2001 to June 2006, we collected residential dust samples using a high volume small surface sampler (HVS3) (Cascade Stack Sampling Systems, Bend, OR). Samples were collected from the room in which the child spent the most awake time (other than the kitchen or bedroom) in the year before diagnosis/reference date, provided that there was a carpet or rug measuring at least 9 ft^2^ that was present before the diagnosis/reference date (otherwise, the next most-used room was sampled). The interviewer typically marked a 4-ft by 6-ft area on the carpet/rug using tape and vacuumed the area in slightly overlapping swaths until approximately 10 ml of dust was collected. Dust samples from household vacuum cleaners were also collected as an additional source of dust for chemical analysis. Concentrations of pesticides and other chemicals in HVS3 dust were highly correlated with concentrations in vacuum cleaner dust [18]; therefore, between July 2006 and November 2007, we switched to collecting dust exclusively from vacuum cleaners, which was less labor-intensive. Collected samples were shipped overnight on ice to Southwest Research Institute (San Antonio, TX), where they were placed in freezers (−12°C). They were subsequently sent to Battelle Memorial Institute (Columbus, OH) and stored in freezers (−20°C) prior to analysis.

### Laboratory analysis

The analysis of dust samples has been described in detail previously [18]. Briefly, dust samples were sieved with a 100-mesh stainless steel sieve to obtain the fine fraction (particles <150 μm) for analysis. We used three extraction methods on separate aliquots from the same overall dust sample (provided there was sufficient dust available) to optimize measurement of all target analytes based on their chemical structures. All insecticides and the herbicides chlorthal and simazine were extracted with hexane/acetone. The herbicides 2,4-D and mecoprop underwent an acid extraction using a 70:30 acetonitrile:phosphate buffer, and piperonyl butoxide was extracted with dichloromethane. Samples were analyzed using gas chromatography/mass spectrometry in multiple ion detection mode. Quality control samples included solvent blanks, duplicate samples, and spiked dust samples. Dust samples were analyzed for 47 pesticides. Of these, 13 had residential uses during the study period and were detected in at least 25% of samples [19,20]: seven insecticides (carbaryl, propoxur, chlorpyrifos, diazinon, cyfluthrin, cypermethrin, permethrin), five herbicides (2,4-dichlorophenoxyacetic acid [2,4-D], chlorthal, dicamba, mecoprop, simazine), and one synergist (piperonyl butoxide).We present results for these 13 pesticides.

### Statistical analysis

Using multivariable linear Tobit regression models for each pesticide, we determined the relationship between pesticide dust concentrations (dependent variables) and ever/never self-reported pest treatments in the past 12 months (independent variables). Tobit regression is an unbiased approach for analyzing measurement data when a substantial proportion of samples are below the limit of detection [19]. We modelled the natural log-transformed pesticide concentrations and estimated the relative change in pesticide concentrations by exponentiating the regression coefficients. We summed concentrations of pesticide isomers (cypermethrin I, II, III, IV, cyfluthrin I, II, III, IV, and cis- and trans-permethrin) because they were highly correlated (*r*_spearman_ > 0.9).

Each insecticide-specific model included the following self-reported pest treatment variables: ants/cockroaches, carpenter ants/termites, fleas/ticks in home, fleas/ticks on pets, flying insects, lawn and garden insects, professional inside treatments, and professional outdoor insect treatments. These pest treatments were moderately correlated (Cramer’s V ranged from 0.15 to 0.77; median = 0.42; Additional file 1: Table S1). We excluded flea/tick shampoo because it was highly correlated with other flea/tick treatment on pets (Cramer’s V > 0.9). We excluded fogger/bomb and indoor plant treatments due to low prevalence (≤5%) and flea/tick collars because we did not measure relevant active ingredients. We combined bees/wasps/hornets and flies/mosquitoes into a “flying insects” category since we lacked data to discriminate active ingredients for these two groups [21]. The models for herbicides included weed treatments by a household member and by a professional as independent variables. A subset of participants who were not asked about professional treatments an early version of the interview (n = 79; 42 cases, 37 controls) were assigned a separate category for the professional treatment variables and retained in the models.

We considered adjustment for a broad range of potential confounders of the relationship between self-reported use and pesticide concentrations in dust including the following demographic and household characteristics: child’s age at diagnosis/reference, child’s sex, child’s race/ethnicity, household income, sampling year, sampling season, when the sampled home was built, whether family members typically removed their shoes upon entering the home, mother’s educational level, number of children residing in the home, residence type (single family home or other), whether a pet lived in the home in the first 2 years of the child’s life (potentially increasing track-in of outdoor pesticide applications [7]), dust sampling method (vacuum or HVS3), frequency of vacuuming, time between diagnosis/reference date and dust collection date, and urbanicity of residential census block (based on population density) [22]. Because all 13 pesticides/synergists were used in both residential and agricultural products, we also considered the effect of nearby agricultural use. As described previously [23,24], agricultural use near the home was determined as the density (mass/unit area) of pesticides applied within a 1250-m buffer around the residence over the 12 months prior to dust collection based on the California Pesticide Use Reporting Database. We included all pest treatment, demographic, household, and agricultural density variables in our initial models. Retaining all pest treatment variables, we removed sequentially the demographic/household characteristics or agricultural density variable with the highest p-value until all remaining covariates had p-values <0.1.

To assess whether the relationship between self-reported pest treatments and concentrations in dust differed by case–control status, we constructed our models separately for cases and controls as well as combined (including case–control status in combined models if p < 0.1). We also tested for interactions between pest treatments and case–control status.

To assess whether the observed associations between treatment for a particular pest and pesticide concentrations in dust were consistent with the composition of commercial pesticide products used by the general public to treat that pest during the time frame of our study, we used information for the year 2000 from the NCI Pesticide Exposure Matrix (http://dceg.cancer.gov/tools/design/pesticide) [21]. This publically available tool uses national data on product sales, active ingredient sales, and pounds of active ingredient from market planning reports to predict the probability that an active ingredient was used for each of 96 scenarios (12 pest types, whether the applicator was a household member [“consumer”] or professional, and 4 timeframes [1976, 1980, 1990, 2000]) [21]. We categorized the probabilities as 0% (active ingredient not listed), 1-9%, 10-19%, and ≥20%.

## Results

The most commonly detected chemicals were the pyrethroid insecticide permethrin (detection rate = 100%), the herbicide 2,4-D (98%), and the synergist piperonyl butoxide (97%) (Table 1). The organophosphate insecticides diazinon and chlorpyrifos and the herbicide mecoprop also had high detection rates (>80%). Four pesticides were detected in <50% of homes: cyfluthrin (25%), cypermethrin (49%), chlorthal (35%), and dicamba (28%). Permethrin had the highest median concentration (1062 ng/g), followed by piperonyl butoxide (151 ng/g) and 2,4-D (102 ng/g).

**Table 1:** **Pesticides concentrations in carpet dust (ng/g) in cases and controls in the Northern California childhood leukemia study, 2001–2007 (n = 583)**

	**N** ^**a**^	**Detection Limit (DL) (ng/g)**	**% > DL**	**25th percentile**	**Median**	**75th percentile**
**Carbamate insecticides**						
Carbaryl	583	2	67	<DL	13.4	43.8
Propoxur	582	5	67	<DL	9.1	33.2
**Organophosphate insecticides**						
Chlorpyrifos	583	5	89	11.9	27.5	73.1
Diazinon	583	2	80	3.1	9.7	31.0
**Pyrethroid insecticides**						
Cyfluthrin	583	80	25	<DL	<DL	93.9
Cypermethrin	583	80	49	<DL	<DL	559
Permethrin	583	4	100	396	1062	4396
**Synergist**						
Piperonyl butoxide	581	4	97	53.0	151	651
**Herbicides**						
2,4-D	571	5	98	34.8	102	419
Chlorthal	583	1	35	<DL	<DL	1.6
Dicamba	572	5	28	<DL	<DL	2.8
Mecoprop	572	5	84	7.6	26.1	111
Simazine	583	2	90	11.3	19.3	32.9

^a^Numbers <583 reflect samples missing due to interferences in chemical analysis or insufficient dust for the appropriate extraction method.

Of the 496 households asked about pest treatment during both the 12 months prior to birth and the 12 months prior to dust collection, most (91%) reported at least one pest treatment in the latter time period, primarily to control ants/cockroaches (71%), weeds by a household member (47%), lawn/garden insects (32%), and flying insects (29%) (Table 2). Pest treatments by a professional were less common; professional outdoor, indoor, and weed treatments in the 12 months prior to dust collection were reported in 28%, 13%, and 11% of households, respectively. The percent agreement for the treatments in the two time periods ranged from 61% to 93%, with a median of 80% (Table 2) and was similar for cases and controls (not shown). The prevalence of self-reported treatments during the 12 months prior to birth was lower compared to the prior 12 months (Table 2). The prevalence of reported use during the last 12 months was slightly higher in controls for most treatment types and was similar between cases and controls in the 12 months prior to birth.

**Table 2:** **Prevalence of self-reported pest treatments 12 months before dust collection and 12 months before birth (n = 496)** ^**a**^

**Treatment**	**% treated 12 months before dust sampling**			**% treated 12 months before birth**			**% agreement between time periods**
	**Overall (n = 496)**	**Cases (n = 241)**	**Controls (n = 255)**	**Overall (n = 496)**	**Cases (n = 241)**	**Controls (n = 255)**	**Overall (n = 496)**
**Insect treatments**							
*Household*							
Ant/Cockroach	71	68	74	47	48	46	61
Lawn/Garden insects	32	29	35	15	12	16	81
Flying insects	29	27	31	14	13	14	77
Fleas/Ticks in home	11	10	13	8	6	9	89
Fleas/Ticks on pets	25	21	29	22	19	25	78
Carpenter Ants/Termites	6	5	8	3	3	2	93
*Professional*							
Professional outdoor^b^	28	28	27	11	11	11	77
Professional indoor^b^	13	11	15	5	5	4	86
**Plant treatments**							
Household weeds	47	44	49	28	24	31	71
Professional weeds^b^	11	13	9	5	8	3	90
**Any treatment**	91	91	92	67	67	66	76

^a^The 496 participants include those who completed questionnaires regarding pest treatments during the 12 months prior to dust collection and the 12 months prior to the child’s birth.

^b^79 participants excluded from calculation because professional questions not included in their interview version.

Tobit regression model estimates and 95% confidence intervals (CIs) for the relative change in insecticides and herbicides are presented separately for cases and controls in Additional file 1: Tables S2 through S5. Results were generally similar. Of the 74 self-reported pest treatment/pesticide associations we evaluated, we observed evidence of interactions (p < 0.1) by case–control status for only 4 (5%) of these relationships. These case control differences in magnitude and statistical significance occurred for carbaryl and fleas/ticks, propoxur and carpenter ants/termites, cyfluthrin and ant/cockroaches, and cypermethrin and professional outdoor treatments. Other differences (p-interaction >0.1) tended to occur for pesticides with low (≤35%) detection rates (e.g. chlorthal) or treatments with low (<15%) prevalence (e.g., carpenter ants/termites, professional indoor treatments).

Table 3 presents the Tobit regression model estimates of the relative change and 95% CIs in insecticide concentrations associated with specific insect treatments in the 12 months prior to dust collection for cases and controls combined. Concentrations of the organophosphates diazinon and chlorpyrifos were respectively 1.5 (95% CI: 1.1, 2.1) and 1.7 (95% CI: 1.3, 2.2) times higher in homes reporting treatment for lawn/garden insects than those without treatments. Chlorpyrifos concentrations were 1.7 (95% CI: 1.0, 2.8) times higher in households reporting treatment of carpenter ants/termites, and diazinon concentrations were 1.5 (95% CI: 1.0, 2.3) times higher in households with professional outdoor treatments, compared to homes without such treatments. The pyrethroids permethrin and cypermethrin were respectively 1.6 and 1.7 times higher in households with treatment for flying insects (95% CI: 1.1, 2.2 and 1.1, 2.8, respectively) than for households without treatments. Permethrin concentrations were 2.3 (95% CI: 1.4, 3.7) times higher in households that reported treating the home for fleas/ticks compared to homes without flea/tick treatment. Concentrations of both cyfluthrin and cypermethrin were respectively 6.8 (95% CI: 3.0, 15) and 2.3 (1.3, 4.1) times higher in homes with professional outdoor treatments compared to those without. Cyfluthrin was associated with treatment for lawn/garden insects 2.3 (95% CI: 1.2, 4.4) and professional indoor treatment 4.0 (1.5, 11). The concentration of the synergist piperonyl butoxide was 1.5 (95% CI: 1.0, 2.1), 2.6 (95% CI: 1.51, 4.4), and 2.0 (1.4, 2.9) times higher in households treating for ant/roaches, fleas/ticks in the home, and fleas/ticks on pets, respectively, compared to households that did not report such treatments. The carbamate insecticides carbaryl and propoxur were not associated with any pest treatments.

**Table 3:** **Relative change in insecticide concentrations with self-reported pest treatments in 12 months before dust collection in cases and controls combined (n = 583)** ^**a**^

	**Relative Change (95% Confidence Interval) in Insecticide Concentrations Relative to Self-reported Pest Treatments (Ever/Never)**							
**Analyte**	**Ants/Roaches**	**Carpenter Ants/ Termites**	**Flea/Tick in Home**	**Flea/Tick on Pets**	**Flying Insects**	**Lawn/Garden Insects**	**Prof. Indoor**	**Prof. Outdoor**
**Carbamates**								
Carbaryl^b^	0.72 (0.44, 1.2)	1.4 (0.53, 3.5)	1.9 (0.89, 3.8)	0.94 (0.56, 1.6)	0.88 (0.53, 1.5)	1.3 (0.81, 2.2)	1.5 (0.66, 3.5)	0.71 (0.37, 1.4)
Propoxur^c^	1.3 (0.91, 1.7)	0.77 (0.42, 1.4)	1.4 (0.88, 2.2)	1.3 (0.94, 1.8)	0.88 (0.64, 1.2)	0.88 (0.64, 1.2)	0.89 (0.52, 1.5)	0.8 (0.54, 1.2)
**Organo-phosphates**								
Chlorpyrifos^d^	0.99 (0.76, 1.3)	**1.7 (1.0, 2.8)**	0.99 (0.67, 1.5)	0.99 (0.76, 1.3)	0.8 (0.61, 1.0)	**1.7 (1.3, 2.2)**	1.5 (0.97, 2.3)	0.85 (0.61, 1.2)
Diazinon^e^	1.0 (0.73, 1.4)	1.4 (0.76, 2.5)	1.5 (0.95, 2.4)	0.87 (0.63, 1.2)	0.92 (0.66, 1.3)	**1.5 (1.1, 2.1)**	1.5 (0.86, 2.5)	**1.5 (1.0, 2.3)**
**Pyrethroids**								
Cyfluthrin^f^	0.70 (0.34, 1.4)	1.2 (0.36, 3.8)	1.5 (0.59, 3.9)	0.95 (0.46, 1.9)	1.1 (0.57, 2.3)	**2.3 (1.2, 4.4)**	**4.0 (1.5, 11)**	**6.8 (3.0, 15)**
Cypermethrin^g^	**2.5 (1.5, 4.2)**	0.91 (0.36, 2.3)	1.9 (0.95, 3.8)	0.65 (0.39, 1.1)	**1.7 (1.1, 2.8)**	1.2 (0.72, 1.9)	0.91 (0.42, 2.0)	**2.3 (1.3, 4.1)**
Permethrin^h^	1.3 (0.97, 1.9)	0.91 (0.49, 1.7)	**2.3 (1.4, 3.7)**	1.2 (0.87, 1.7)	**1.6 (1.1, 2.2)**	0.74 (0.54, 1.0)	1.6 (0.95, 2.8)	0.97 (0.64, 1.5)
**Synergist**								
Piperonyl butoxide^i^	**1.5 (1.0, 2.1)**	1.2 (0.61, 2.5)	**2.6 (1.5, 4.4)**	**2.0 (1.4, 2.9)**	1.2 (0.85, 1.8)	0.70 (0.49, 1.0)	1.6 (0.85, 2.8)	0.74 (0.46, 1.2)

^a^Bold typface indicates 95% confidence interval does not include 1.

^b^Adjusted for ethnicity, when home built, interview year, duration between reference/diagnosis and sampling.

^c^Adjusted for when home built, interview year, urbanicity, duration between reference/diagnosis and sampling.

^d^Adjusted for income, when home built, interview year, density of agricultural use.

^e^Adjusted for season, maternal education, interview year.

^f^Adjusted for ethnicity, season, when home built, pets in home.

^g^Adjusted for when home built, frequency of vacuuming, maternal education, pets in home, # children in homes.

^h^Adjusted for shoe removal, urbanicity, # children in home, interview year.

^i^Adjusted for ethnicity, income, interview year.

Reports of household weed treatment in the previous 12 months significantly predicted 2.8 times higher concentrations of 2,4-D (95% CI: 2.1, 3.7), 1.9 (95% CI: 1.4, 2.6) times higher concentrations of dicamba, and 2.2 (95% CI: 1.6, 3.1) times higher concentrations of mecoprop (Table 4). No link with household weed treatment was observed for chlorthal or simazine, but agricultural use was a significant predictor of these two herbicides (not shown). Professional weed treatments were inversely associated with 2,4-D (0.54, 95% CI: 0.34, 0.84) and mecoprop (0.60, 95% CI: 0.37, 0.99). A case–control indicator variable evaluated in all combined models was only included in the model for chlorthal (p < 0.1) although the test for interaction with household weed treatment was not significant (p-interaction = 0.4).

**Table 4:** **Relative change in herbicide concentrations with self-reported weed treatments in 12 months before dust collection in cases and controls combined (n = 583)** ^**a**^

	**Relative change (95% confidence interval) in herbicide concentrations relative to self-reported weed treatments (ever/never)**	
**Analyte**	**Weed treatment by household member**	**Weed treatment by professional**
2,4-D^b^	**2.8 (2.1, 3.7)**	**0.54 (0.34, 0.84)**
Chlorthal^c^	1.3 (0.88, 1.9)	0.72 (0.39, 1.3)
Dicamba^d^	**1.9 (1.4, 2.6)**	0.90 (0.56, 1.5)
Mecoprop^e^	**2.2 (1.6, 3.1)**	**0.60 (0.37, 0.99)**
Simazine^f^	1.1 (0.85, 1.3)	1.1 (0.75, 1.5)

^a^Bold typface indicates 95% confidence interval does not include 1.

^b^Adjusted for child ethnicity, season, residence type, frequency of vacuuming, urbanicity, interview year.

^c^Adjusted for child ethnicity, income, when residence built, interview year, density of agricultural use.

^d^Adjusted for mother’s education, pets in home, interview year.

^e^Adjusted for ethnicity, income, residence built, residence type, shoe removal, vacuum frequency, urbanicity, interview year.

^f^Adjusted for income, residence type, maternal education, interview year, density of agricultural use.

Six insecticides had a probability of use of ≥20% for one or more insect type according to the NCI Pesticide Exposure Matrix (Table 5). Five of these insect/insecticide pairs were significantly associated in our models (cases and controls combined): lawn/garden insects and chloryprifos, lawn/garden insects and diazinon, fleas/ticks and permethrin, flying insects and permethrin, termites and chlorpyrifos (Table 5). Of the 18 insect-insecticide combinations in Table 5 with probabilities of 0% (i.e., insecticide not listed for that pest in the Pesticide Exposure Matrix), only one (lawn and garden insects/cyfluthrin) was significantly associated in our data. Of the five instances of weed/herbicide combinations with probabilities of ≥20% (Table 6), three exhibited positive associations in our models (cases and controls combined): household weeds and 2,4-D, dicamba, and mecoprop. The four weed-herbicide combinations with <10% probability were not significantly associated.

**Table 5:** **Expected probabilities** ^**a**^ **(exp) of insecticide use and observed** ^**b**^ **associations (obs) between dust concentrations and self-reported insect treatments**

	**Ants/roaches**		**Termites**		**Fleas/ticks in the home**		**Fleas/ticks on pets**		**Flying insects**		**Lawn/garden insects**		**Professional indoor insects**		**Professional outdoor insects**	
	**Prob %**	**Obs** ^**c,d**^	**Prob %**	**Obs** ^**c,d**^	**Prob %**	**Obs** ^**c,d**^	**Prob %**	**Obs** ^**c,d**^	**Prob %**	**Obs** ^**c,d**^	**Prob %**	**Obs** ^**c,d**^	**Prob %**	**Obs** ^**c,d**^	**Prob %**	**Obs** ^**c,d**^
Carbaryl	1-9		0		1-9		1-9		1-9		1-9		0		1-9	
Propoxur	1-9		0		1-9		0		1-9		0		0		0	
Chlorpyrifos	10-19		≥20	+	10-19		0		1-9		≥20	+	1-9		10-19	
Diazinon	1-9		0		10-19		0		1-9		≥20	+	1-9		1-9	+
Cyfluthrin	1-9		0		1-9		0		1-9		0	+	1-9	+	1-9	+
Cypermethrin	1-9	+	1-9		0		0		1-9	+	0		≥20		0	
Permethrin	10-19		1-9		≥20	+	10-19		≥20	+	0		1-9		1-9	

^a^Reflects probability of use of insecticide active ingredient for year 2000, based on NCI Pesticide Exposure Matrix [21].

^b^Observed reflects statistical significance of association from multivariable regression models presented in Table 3.

^c^ +, positive association, 95% confidence interval (CI) does not include 1.

^d^Empty cell reflects no association, 95% CI includes 1.

**Table 6:** **Expected probabilities** ^**a**^ **(exp) of herbicide use and observed** ^**b**^ **associations (obs) between dust concentrations and self-reported weed treatments**

**Herbicides**	**Weed treatment by household member/consumer**		**Weed treatment by professional**	
	**Prob %**	**Obs** ^**c,d,e**^	**Prob %**	**Obs** ^**c,d,e**^
2,4-D	≥20	+	≥20	**–**
Chlorthal	1-9		0	
Dicamba	≥20	+	≥20	
Mecoprop	≥20	+	10-19	**–**
Simazine	0		1-9	

^a^Reflects probability of use of herbicide active ingredient based on NCI Pesticide Exposure Matrix [21].

^b^Observed reflects statistical significance of association from multivariable regression models presented in Table 4.

^c^ +, positive association, 95% confidence interval (CI) does not include 1.

^d^ -, negative association, 95% CI does not include 1.

^e^Empty cell reflects no association, 95% CI includes 1.

To explore whether self-reported pest treatment during the 12 months prior to birth (“prenatal”), a potentially etiologically relevant time period for childhood leukemia, predicted post-diagnosis/reference concentrations of pesticides in dust, adjusted for treatments in the 12 months prior to sampling, we evaluated the association between prenatal pest treatments and pesticide concentrations by adding terms for the prenatal treatments into the final models for use in the previous 12 months (not shown). We observed several statistically significant associations between prenatal pest treatments and concentrations of certain pesticides in dust, such as carpenter ants/termites and chlorpyrifos (2.4, 95% CI: 1.1, 5.5), fleas/ticks on pets and permethrin (1.7, 95% CI: 1.1, 2.6), professional indoor treatments and concentrations of chlorpyrifos (2.1, 95% CI: 1.0, 4.3), carpenter ants/termites and piperonyl butoxide 3.8 (95% CI: 1.3, 11) and household treatment of weeds and mecoprop (1.6, 95% CI: 1.1, 2.4).

## Discussion

We examined the relationship between two approaches for assessing residential pesticide exposure: questions about self-reported treatment for specific pests and measurements of active ingredients in carpet dust. We found that these two exposure assessment approaches were generally consistent with one another, lending credibility to both. Similar relationships between common household pest treatments and pesticides concentrations in homes between cases and controls suggests that recall bias may be minimal. Our findings support the validity of studies of pesticide exposure and childhood leukemia risk, which are based primarily on self-report.

Insecticides with a high predicted probability (≥20%) of being present in a product used to treat a specific type of insect were consistently found at higher concentrations in households reporting treatment for that insect. The NCI Pesticide Exposure Matrix was a useful tool for evaluating our observed associations. However, because its most current year was 2000, it did not capture changing chemical formulations during the study period (2001–2007). For example, residential uses of chlorpyrifos and diazinon were restricted in and after the year 2000 [25,26], and were likely replaced by the pyrethroids [27]. In addition, we could not comment on the plausibility of associations with the synergist piperonyl butoxide because the matrix covers only active ingredients. The laboratory methods we used were not suitable for some residential-use insecticides with a high predicted probability of use, such as fipronil and imidacloprid in flea/tick treatments.

For the herbicides, reported treatment of weeds by a household member was associated with higher concentrations of the herbicides dicamba, 2,4-D, and mecoprop, all with high probabilities of being found in a home and garden weed product used by consumers. Density of agricultural use was a significant predictor of both simazine and chlorthal, suggesting that levels of these chemicals in the study homes were more related to agricultural than household usage. Professional weed treatments were inversely or not associated with any herbicides, even though 2,4-D and dicamba had high probabilities of being in a professional weed product. Study participants and professionals might have been using herbicides that were not measured in our study, such as glyphosate (the active ingredient in the popular “Round-up” products), which had a 10% probability of use for consumer and professional weed treatments in 2000 [21] and increasing residential use after 2000 [28,29]. Other (unmeasured) factors that could have affected the associations for weed and insect pests include cleaning practices, permeability of the house to pesticide drift from outdoors, behaviors leading to track-in, and other sources. We also lacked information on amount of active ingredients applied and how recently the application took place.

Our results were mostly consistent with previous studies with detailed questions about home and garden pest treatments and pesticide measurements in dust. Colt et al. [5], who studied controls in an adult non-Hodgkin lymphoma study, measured six of the same pesticides (carbaryl, propoxur, chlorpyrifos, permethrin, 2,4-D, and dicamba) in dust samples collected from 1999–2001 from 513 homes in four U.S. locations. They observed two of the six significant associations we observed with these active ingredients: flea/tick treatment and permethrin, and weed treatment and 2,4-D. Additionally, they reported an association between chlorpyrifos and “crawling insects” whereas we observed an association with chlorpyrifos and lawn/garden insects. Differences in associations could be related to differences in wording of pest treatment questions, time periods, and geographic regions. In Deziel et al. [12], repeated dust samples collected from 21 homes of healthy adults in Fresno, California (2003–2005) were analyzed for ten chemicals in common with our study. Two associations in our study were consistent with this temporally similar population: diazinon and lawn/garden insects and cypermethrin and professional outdoor insects [12]. In the Mexican Immigration to California: Agricultural Safety and Acculturation (MICASA) Study, a prospective study of farmworker families in Mendota, California, five pyrethroids (including permethrin and cypermethrin) were measured in 55 farmworker homes in 2009 [30]. Use of outdoor pesticide sprays around the home was correlated with levels of cypermethrin in the house dust, similar to the relationship we observed with “professional outdoor” insect treatment and cypermethrin.

Studies that asked broader questions about residential pesticide use generally observed null or mostly null associations with pesticide levels in dust. In the Minnesota Children’s Pesticide Exposure Study, six pesticide-use questions were not predictive of surface wipe loadings in urban and non-urban homes in Minnesota [31]. Differences could be due to collection methods (surface sample versus bulk dust) and specificity in questions (e.g., questions about individual pests vs. any indoor pests). In the NHEXAS-Maryland Study, conducted in the city of Baltimore from 1995–1996, questions about number, timing, and location of pesticide use were not associated with chlorpyrifos concentrations in dust [32]. They did not ask about treatments for specific pests. Our study adds to this body of literature suggesting that specific questions about the type of pest treated can be good predictors of concentrations of specific pesticides in homes.

A question of interest was whether post-diagnosis dust samples are adequate to characterize exposure before diagnosis and especially during the potentially etiologically relevant prenatal period. In general, we observed that reported pest treatments during the last 12 months and 12 months before birth exhibited high percent agreement. Additionally, due to their persistence indoors, measurements of pesticides in carpet dust samples are likely to reflect pesticide use over both time periods. As a result, we were unable to disentangle the independent contribution of prenatal pesticide usage to post-diagnosis pesticide concentrations in dust samples. However, the high agreement between pest treatment practices in both time periods as well as our previous research demonstrating moderate to high repeatability in pesticide concentrations measured in dust over time [12], are supportive of using post-diagnosis dust samples to characterize exposures before diagnosis.

Key strengths of this analysis include the relatively large sample size and inclusion of a range insecticides and herbicides representing different chemical classes. In addition, the evaluation of potential case–control differences in pest treatment reporting is highly relevant to epidemiologic studies. One limitation not previously stated is that our findings may not be generalizable to the full NCCLS population or other populations because eligibility for the dust sampling was limited to more residentially stable families. Also, due to changes in the questionnaires, we were missing data for some participants for the professional pest treatment questions and treatments during the earlier time periods. Because our analysis involved many comparisons between various pest treatments and pesticides, it is possible that some of the associations observed were due to chance. Accuracy of reporting may have increased if the father also answered the questions regarding home and garden pest treatments. Future studies could incorporate additional details, such as frequency of use, dates of application, or product brand names to potentially improve associations between self-reports and concentrations of specific pesticide active ingredients in the dust.

## Conclusions

Retrospective assessment of pesticide exposure during critical time windows for diseases such as childhood leukemia is challenging. We observed positive associations between self-reported treatment for specific pests and levels of most active ingredients measured in dust samples with generally similar findings for cases and controls. Our analysis supports the utility of both methods of exposure assessment and suggests that recall bias of past pesticide treatments is minimal. Each method has unique strengths. While measurements of carpet dust provide information on active ingredients, only interview data can provide information on household behaviors such as rooms occupied by children, timing of pesticide use, and other covariates important for evaluating pesticide exposure.

## Additional file

Additional file 1: Table S1. Cramer’s V values for correlations among pest treatments reported in the 12 months prior to dust collection (n = 583). **Table S2.** Relative change in insecticide concentrations with self-reported insect treatments in 12 months before dust collection among cases (n = 277). **Table S3.** Relative change in insecticide concentrations with self-reported insect treatments in 12 months before dust collection among controls (n = 306). **Table S4.** Relative change in herbicide concentrations with self-reported weed treatments in 12 months before dust collection among cases (n = 277). **Table S5.** Relative change in herbicide concentrations with self-reported weed treatments in 12 months before dust collection among controls (n = 306).
