# Supplementary material for: Associations between self-reported pest treatments and pesticide concentrations in carpet dust
Source: Environ Health. 2015 Mar 25;14:27. doi: 10.1186/s12940-015-0015-x (PMC4374193; doi:10.1186/s12940-015-0015-x)
Supplement: Additional file 1: Table S1. — Cramer’s V values for correlations among pest treatments reported in the 12 months prior to dust collection (n = 583). Table S2. Relative change in insecticide concentrations with self-reported insect treatments in 12 months before dust collection among cases (n = 277). Table S3. Relative change in insecticide concentrations with self-reported insect treatments in 12 months before dust collection among controls (n = 306). Table S4. Relative change in herbicide concentrations with self-reported weed treatments in 12 months before dust collection among cases (n = 277). Table S5. Relative change in herbicide concentrations with self-reported weed treatments in 12 months before dust collection among controls (n = 306). [file 12940_2015_15_MOESM1_ESM.docx]

**Supplemental Tables**

Associations between Self-Reported Pest Treatments and Pesticide Concentrations in Carpet Dust

Nicole C. Deziel, Joanne S. Colt, Erin E. Kent, Robert B. Gunier, Peggy Reynolds, Benjamin Booth, Catherine Metayer, Mary H. Ward

**Supplemental Table S1.** Cramer's V values for correlations among pest treatments reported in the 12 months prior to dust collection (n=583).

**Supplemental Table S2.** Relative change in insecticide concentrations with self-reported insect treatments in 12 months before dust collection among cases (n=277).

**Supplemental Table S3.** Relative change in insecticide concentrations with self-reported insect treatments in 12 months before dust collection among controls (n=306).

**Supplemental Table S4.** Relative change in herbicide concentrations with self-reported weed treatments in 12 months before dust collection among cases (n=277).

**Supplemental Table S5.** Relative change in herbicide concentrations with self-reported weed treatments in 12 months before dust collection among controls (n=306).

| Supplemental Table S1. Cramer's V values for correlation among pest treatments reported in the 12 months prior to dust collection (n=583). | | | | | | | | | | |
| --- | --- | --- | --- | --- | --- | --- | --- | --- | --- | --- |
|  | Ants/ Cockroach | Carpenter Ants/ Termites | Flea/Tick in Home | Flea/Tick on Pets | Flying Insects | Lawn/ Garden Insects | Prof. Outdoor | Prof. Indoor | Lawn/ Garden Weeds | Prof. Weeds |
| Ants/Cockroach | 1.00 |  |  |  |  |  |  |  |  |  |
| Carpenter Ants/Termites | 0.54 | 1.00 |  |  |  |  |  |  |  |  |
| Fleas/Ticks in Home | 0.51 | 0.67 | 1.00 |  |  |  |  |  |  |  |
| Fleas/Ticks on Pets | 0.50 | 0.66 | 0.63 | 1.00 |  |  |  |  |  |  |
| Flying Insects | 0.42 | 0.54 | 0.51 | 0.51 | 1.00 |  |  |  |  |  |
| Lawn/Garden Insects | 0.48 | 0.54 | 0.51 | 0.50 | 0.42 | 1.00 |  |  |  |  |
| Professional Outdoor | 0.20 | 0.23 | 0.18 | 0.19 | 0.17 | 0.15 | 1.00 |  |  |  |
| Professional Indoor | 0.21 | 0.22 | 0.18 | 0.19 | 0.17 | 0.16 | 0.77 | 1.00 |  |  |
| Lawn/Garden Weeds | 0.46 | 0.59 | 0.63 | 0.56 | 0.45 | 0.47 | 0.17 | 0.18 | 1.00 |  |
| Professional Weeds | 0.18 | 0.19 | 0.18 | 0.19 | 0.16 | 0.23 | 0.65 | 0.69 | 0.29 | 1.00 |

| Supplemental Table S2 Relative change in insecticide concentrations with self-reported insect treatments in 12 months before dust collection among cases (n=277). | | | | | | | | | |
| --- | --- | --- | --- | --- | --- | --- | --- | --- | --- |
|  | Relative Change (95% Confidence Interval) | | | | | | | | |
| **Analyte** | **Ants/ Cockroaches** | **Carpenter Ants/ Termites** | **Flea/Tick in Home** | **Flea/Tick on Pets** | **Flying  Insects** | **Lawn/Garden Insects** | **Professional Indoor** | **Professional Outdoor** |  |
| **Carbamates** |  |  |  |  |  |  |  |  |  |
| Carbaryl^a^ | 1.0 (0.5, 1.9) | 0.6 (0.1, 3.4) | 1.2 (0.37, 4.0)* | 0.8 (0.3, 1.7) | 1.0 (0.5, 2.1) | 1.5 (0.7, 3.1) | 2.0 (0.6, 7.5) | 0.8 (0.3, 2.1) |  |
| Propoxur^b^ | 1.4 (0.9, 2.2) | **0.27 (0.1, 0.8)*** | 1.5 (0.8, 3.1) | 1.6 (1.0, 2.6) | 1.2 (0.7, 1.8) | 0.9 (0.6, 1.4) | 0.8 (0.3, 1.7) | 0.7 (0.4, 1.3) |  |
| **Organophosphates** |  |  |  |  |  |  |  |  |  |
| Chlorpyrifos^c^ | 1.2 (0.8, 1.7) | 1.2 (0.5, 3.1) | 1.0 (0.5, 1.9) | 1.3 (0.8, 2.0) | 1.1 (0.8, 1.7) | **1.5 (1.0, 2.3)** | 1.6 (0.8, 3.3) | 0.6 (0.4, 1.0) |  |
| Diazinon^d^ | 1.0 (0.7, 1.6) | 1.3 (0.4, 3.8) | 1.7 (0.8, 3.7) | 0.8 (0.4, 1.3) | 1.0 (0.6, 1.7) | **1.6 (1.0, 2.5)** | 1.0 (0.5, 2.3) | 1.7 (0.9, 3.0) |  |
| **Pyrethroids** |  |  |  |  |  |  |  |  |  |
| Cyfluthrin^e^ | **0.3 (0.1, 0.9)*** | 1.2 (0.2, 9.5) | 1.4 (0.3, 5.8) | 0.5 (0.2, 1.6) | 1.1 (0.4, 2.9) | **4.2 (1.6, 11.1)** | **17.9 (4.1, 77.5)** | **4.5 (1.5, 13.9)** |  |
| Cypermethrin^f^ | 2.4 (1.3, 4.7) | 0.4 (0.1, 1.8) | 2.4 (0.9, 6.5) | 0.8 (0.4, 1.7) | 1.3 (0.7, 2.5) | 1.2 (0.6, 2.4) | 1.6 (0.6, 4.4) | **3.3 (1.5, 7.0)*** |  |
| Permethrin^g^ | 1.2 (0.8, 1.8) | 0.8 (0.3, 2.5) | **3.2 (1.5, 6.7)** | 1.3 (0.8, 2.2) | 1.1 (0.6, 1.7) | 0.9 (0.5, 1.4) | 2.2 (1.0, 5.1) | 0.8 (0.5, 1.5) |  |
| **Synergist** |  |  |  |  |  |  |  |  |  |
| Piperonyl butoxide^h^ | **1.8 (1.1, 2.8)** | 1.0 (0.3, 3.5) | **2.3 (1.1, 5.2)** | 1.4 (0.8, 2.5) | 1.5 (0.9, 2.5) | 0.8 (0.5, 1.3) | 1.0 (0.4, 2.5) | 0.7 (0.3, 1.2) |  |
| *p<0.1 for test for interaction between cases and controls ^a^ Adjusted for ethnicity, when residence built, interview year ^b^ Adjusted for when residence built, urbanicity, # children in residence, interview year  ^c^ Adjusted for when residence built, pets in home, duration between reference/diagnosis and sampling, density of agricultural use ^d^ Adjusted for child’s age, income, interview year  ^e^ Adjusted for ethnicity, interview season, year residence built, pets in home, # children in residence f Adjusted for child’s age, when residence built, shoe removal, urbanicity, maternal education, # children in residence, pest in home, interview year ^g^ Adjusted for shoe removal, urbanicity, # children in residence, interview year ^h^ Adjusted for child’s sex, child’s age, residence type, urbanicity, maternal education, # children in residence | | | | | | | | | |

| Supplemental Table S3. Relative change in insecticide concentrations with self-reported insect treatments (ever/never) in 12 months before dust collection among controls (n=306). | | | | | | | | |
| --- | --- | --- | --- | --- | --- | --- | --- | --- |
|  | Relative Change (95% Confidence Interval) | | | | | | | |
| **Analyte** | **Ants/ Cockroaches** | **Carpenter Ants/ Termites** | **Flea/Tick in Home** | **Flea/Tick on Pets** | **Flying  Insects** | **Lawn/Garden Insects** | **Professional Indoor** | **Professional Outdoor** |
| **Carbamates** |  |  |  |  |  |  |  |  |
| Carbaryl^a^ | 0.5 (0.2, 1.0) | 2.4 (0.8, 7.4) | **2.8 (1.1, 7.3)*** | 1.0 (0.5, 1.9) | 0.9 (0.4, 1.6) | 1.5 (0.8, 2.8) | 0.9 (0.3, 2.8) | 0.8 (0.3, 1.9) |
| Propoxur^b^ | 1.1 (0.7, 1.8) | 1.1 (0.7, 1.8)* | 1.4 (0.7, 3.06) | 1.2 (0.8, 1.8) | 0.7 (0.5, 1.1) | 0.7 (0.4, 1.1) | 0.9 (0.5, 1.6) | 0.8 (0.4, 1.6) |
| **Organophosphates** |  |  |  |  |  |  |  |  |
| Chlorpyrifos^c^ | 0.8 (0.6, 1.2) | **2.1 (1.2, 3.7)** | 1.1 (0.7, 1.6) | 0.9 (0.6, 1.2) | 0.7 (0.5, 1.0) | **1.6 (1.2, 2.2)** | 1.1 (0.7, 1.6) | 1.3 (0.8, 2.1) |
| Diazinon^d^ | 1.0 (0.7, 1.6) | 1.3 (0.6, 2.6) | 1.3 (0.7, 2.3) | 1.1 (0.8, 1.7) | 0.9 (0.6, 1.3) | **1.6 (1.1, 2.4)** | **2.0 (1.0, 4.0)** | 1.5 (0.9, 2.6) |
| **Pyrethroids** |  |  |  |  |  |  |  |  |
| Cyfluthrin^e^ | 1.4 (0.5, 4.1)* | 1.6 (0.4, 7.1) | 1.4 (0.4, 5.0) | 1.5 (0.6, 3.7) | 1.0 (0.4, 2.6) | 1.4 (0.6, 3.7) | 1.4 (0.4, 5.3) | **9.5 (3.0, 30)** |
| Cypermethrin^f^ | **2.4 (1.1, 5.2)** | 1.7 (0.5, 5.4) | 2.5 (1.0, 6.3) | 0.7 (0.4, 1.5) | **2.3 (1.2, 4.5)** | 0.8 (0.4, 1.6) | 0.6 (0.2, 1.6) | 1.5 (0.6, 3.6)* |
| Permethrin^g^ | 1.5 (1.0, 2.3) | 1.0 (0.5, 2.0) | **2.2 (1.2, 4.1)** | 1.0 (0.7, 1.6) | **2.0 (1.3, 3.0)** | 0.7 (0.4, 1.0) | 1.3 (0.6, 2.6) | 1.0 (0.6, 1.8) |
| **Synergist** |  |  |  |  |  |  |  |  |
| Piperonyl butoxide^h^ | 1.3 (0.8, 2.1) | 1.32 (0.6, 3.1) | **2.5 (1.2, 4.9)** | **2.3 (1.4, 3.8)** | 1.2 (0.7, 1.9) | 0.7 (0.4, 1.1) | 2.0 (0.9, 4.4) | 0.9 (0.5, 1.6) |
| ^*^p<0.1 for test for interaction between cases and controls ^a^ Adjusted for ethnicity, pets in home, interview year, duration between reference/diagnosis and sampling ^b^ Adjusted for when residence built, vacuum frequency, urbanicity, # children in residence, interview year, duration between reference/diagnosis and sampling  ^c^ Adjusted for child’s age, income, when residence built, vacuum frequency, interview year, density of agricultural use ^d^ Adjusted for child’s age, interview season, urbanicity, maternal education, interview year  ^e^ Adjusted for ethnicity, when residence built, pets in home, interview year  ^f^ Adjusted for child’s sex, ethnicity, income, when residence built, shoe removal, vacuum frequency, maternal education, pets in home  ^g^ Adjusted for interview year, duration between reference/diagnosis and sampling, pets in home  ^h^ Adjusted for ethnicity, income, year interview, pets in home, density of agricultural use | | | | | | | | |

| Supplemental Table S4. Relative change in herbicide concentrations with self-reported weed treatments in 12 months before dust collection among cases (n=277). | | |
| --- | --- | --- |
| **Analyte** | **Weed Treatment by**  **Household Member** | **Weed Treatment by Professional** |
| 2,4-D^a^ | **2.8 (1.8, 4.2)** | 0.7 (0.4, 1.3) |
| Chlorthal^b^ | 0.9 (0.5, 1.4) | 1.0 (0.5, 2.0) |
| Dicamba^c^ | **1.7 (1.0, 2.7)** | 1.6 (0.8, 3.1) |
| Mecoprop^d^ | **2.9 (1.9, 4.4)** | 0.8 (0.4, 1.4) |
| Simazine^e^ | 1.2 (0.9, 1.6) | 0.9 (0.6, 1.4) |
| ^a^ Adjusted for residence type, vacuum frequency, urbanicity, maternal education ^b^ Adjusted for child’s age, ethnicity, # children in residence, interview year, density of agricultural use, duration between reference/diagnosis and sampling  ^c^ Adjusted for season, maternal education, interview year ^d^ Adjusted for income, residence type, vacuum frequency, urbanicity, maternal education, interview year ^e^ Adjusted for income, residence type, interview year, density of agricultural use | | |

| Supplemental Table S5. Relative change in herbicide concentrations with self-reported weed treatments in 12 months before dust collection among controls (n=306). | | |
| --- | --- | --- |
| **Analyte** | **Weed Treatment by Household Member** | **Weed Treatment by Professional** |
| 2,4-D^a^ | **2.9 (2.0, 4.1)** | **0.4 (0.2, 0.7)** |
| Chlorthal^b^ | **2.0 (1.1, 3.6)** | 1.0 (0.4, 2.8) |
| Dicamba^c^ | **2.1 (1.5, 3.1)** | 0.6 (0.3, 1.2) |
| Mecoprop^d^ | **2.2 (1.4, 3.4)** | **0.4 (0.2, 0.9)** |
| Simazine^e^ | 0.9 (0.7, 1.3) | 1.3 (0.8, 2.1) |
| ^a^ Adjusted for ethnicity, season, when residence built, vacuum frequency, urbanicity ^b^ Adjusted for income, shoe removal, when residence built, maternal education, # children in residence, interview year, density of agricultural use, duration between reference/diagnosis and sampling  ^c^ Adjusted for season, vacuum frequency, maternal education, pets in home, interview year, density of agricultural use ^d^ Adjusted for child’s age, ethnicity, season, when residence built, residence type, shoe removal, urbanicity ^e^ Adjusted for income, when residence built, maternal education, interview year, density of agricultural use | | |
